# Analysis of Complications of a Neglected Disease: 13 Years of Experience with Liver Hydatid Cysts in a High-Volume Hospital

**DOI:** 10.3390/medicina60101696

**Published:** 2024-10-15

**Authors:** Mustafa Azizoğlu, Bahattin Aydogdu, Tahsin Onat Kamci, Serkan Arslan, Erol Basuguy, Salim Bilici, Mehmet Hanifi Okur

**Affiliations:** 1 Department of Pediatric Surgery, Medical School, Dicle University, Diyarbakır 21280, Turkey; bahattinaydogdu@hotmail.com (B.A.); onatkamci@gmail.com (T.O.K.); drserkanarslan@hotmail.com (S.A.); erbas.80@hotmail.com (E.B.); salbilici@hotmail.com (S.B.); okurmh@gmail.com (M.H.O.); 2 Department of Stem Cell and Tissue Engineering, Istinye University, Istanbul 34460, Turkey; 3 Pediatric Surgery Clinic, Esenyurt State Hospital, Istanbul 34517, Turkey

**Keywords:** liver hydatid cyst, *Echinococcus granulosus*, cysto-biliary fistula, complications analysis

## Abstract

*Background and Objectives*: The aim of this study was to evaluate the clinical presentation, treatment outcomes, and complications associated with hepatic hydatid cysts in a pediatric population. *Materials and Methods*: This retrospective study analyzed 214 pediatric patients with liver hydatid cysts, focusing on clinical presentation, treatment outcomes, and associated complications. Patients were classified based on treatment modality, including non-operative management with albendazole, PAIR, and surgical intervention. This study compared cyst characteristics, recurrence rates, and complications such as cysto-biliary fistulas. *Results*: Among the patients, 68% (*n* = 145) had a single cyst and 86% (*n* = 184) were found to have isolated liver cysts. No significant statistical difference was observed between Group 1 and Group 2 in terms of age, gender, and basic laboratory values and general characteristics of the cysts, such as the lobe where the cyst was located, involvement of multiple organs, number of cysts, the state of cyst rupture, and recurrence; no statistically significant difference was found between the groups (*p* > 0.05 for each comparison). Cyst rupture incidence was 6%, and the average incidence of recurrence was 2%, with a surgical recurrence incidence of 3%. A total of 37 patients had a laparotomy, while 7 had laparoscopic surgery. In total, capitonnage was performed in 68 patients, omentopexy in 4, and cystostomy in 6. Consequently, among the treated patients (PAIR + surgery), the incidence of cysto-biliary fistula was 11%, anaphylaxis was 2%, surgical recurrence was 3%, and the incidence of reoperation (Clavien–Dindo ≥ 3) was 6%. The average follow-up period was 72 months, during which no mortality was observed. *Conclusions*: We identified key clinical outcomes related to both non-surgical treatments (cyst rupture and recurrence) and surgical groups (cysto-biliary fistulas, anaphylaxis, the need for reoperation, rupture, and recurrence).

## 1. Introduction

Hydatid disease of the liver, also known as cystic echinococcosis (CE), is a cystic condition caused by the *Echinococcus granulosus* parasite in the liver. This parasite is transmitted to humans through the feces of infected dogs. Humans ingest the parasite’s eggs through contaminated food or water. Once ingested, these eggs produce larvae in the intestines, which can develop into cysts. These cysts can grow in the liver and may reach significant sizes over time [1].

It is estimated that there are 2–3 million global cases of the disease. However, the overall prevalence is likely underestimated due to a lack of comprehensive epidemiological studies in all endemic regions. *Echinococcus granulosus* s.l. is prevalent in countries such as India, Australia, Turkey, China, South America, and several Middle Eastern and Eastern European nations, with incidence rates reaching up to 50 per 100,000 person-years and an estimated prevalence of 10% in highly endemic areas. Factors such as low socioeconomic status and unsanitary animal slaughtering practices contribute to the increased incidence in these regions [2,3].

In many cases, liver hydatid disease is asymptomatic. However, large cysts can impair liver functions, leading to symptoms such as abdominal pain, loss of appetite, jaundice, and abdominal swelling. Imaging techniques like ultrasound and CT scans are commonly used for diagnosis. Ultrasound is especially popular for identifying the location, number, size, and wall thickness of the cysts, as well as providing information about the cysts’ fluid content and any solid areas within them [4,5].

The three main treatment methods for liver hydatid disease are medical treatment, PAIR (Puncture, Aspiration, Injection, Re-aspiration), and surgery. Initially, open surgery was the standard treatment for liver cysts, but the development of effective chemotherapy, such as albendazole, has made PAIR the preferred method [2,6,7]. However, surgery remains the primary method for treating extrahepatic CE [4,5,8]. Albendazole, often used in conjunction with adjuvant therapy, has shown fewer complications, higher cure rates, and lower recurrence rates than surgical intervention, making it a frequently used treatment for liver hydatid disease [9]. Albendazole is particularly recommended in cases where there is a higher risk of cyst rupture, infection, and allergic reactions [9]. It is also suggested for patients with multiple organ involvement or small, uncomplicated cysts, as well as asymptomatic patients. Some studies indicate that up to 25% of patients treated solely with albendazole may experience a recurrence. Both recurrence and spontaneous resolution are possible outcomes, but younger cysts and those in younger individuals may be more responsive to albendazole due to higher metabolic activity [4,8,10,11].

The aim of this study was to evaluate the clinical presentation, treatment outcomes, and complications associated with hepatic hydatid cysts in a pediatric population.

## 2. Materials and Methods

### 2.1. Patients and Groups

This study presents a retrospective analysis conducted on a cohort of 214 patients diagnosed with hydatid disease of the liver at Dicle University Pediatric Surgery Clinic (Turkey) over a twelve-year period from 1 January 2010 to 30 December 2022. The patients’ files were enrolled retrospectively. The patients included in this study were divided into two distinct groups based on their treatment approach. Group 1 consisted of patients who received medical treatment or were placed under observation (wait-and-watch approach), while Group 2 comprised patients who underwent Percutaneous Aspiration, Injection, and Re-aspiration (PAIR) or surgical intervention. The evaluation focused on a range of demographic and clinical parameters, including age, presenting symptoms, and diagnostic and treatment histories.

### 2.2. Ethical Approval

The ethical committee approval number was obtained from the Dicle University non-interventional ethical committee with number/date: 48/17 January 2023.

### 2.3. Inclusion Criteria

Patients aged 0–18 years, diagnosed with hydatid cysts at our clinic, who have undergone diagnosis and treatment, and whose retrospective data are organized and consistent, were included in the study.

### 2.4. Exclusion Criteria

Patients older than 18 years, those whose retrospective data are not regular and reliable, and those initially diagnosed at our clinic but followed up at another hospital in subsequent periods were excluded from the study.

### 2.5. Definition of the Grade of CE

CE is primarily diagnosed using imaging techniques such as ultrasound and computed tomography (CT) [1,2,4,10,12,13]. Ultrasound is the most commonly used method and is considered the gold standard for diagnosing liver CE. It provides information about the number of complications, location, internal structure, and presence of the cyst, with approximately 95% sensitivity and 90% specificity [2,3,13,14,15]. Various classifications based on characteristic ultrasound findings have been developed, with the best-known being the Gharbi classification and the World Health Organization (WHO) classification for CE [16]. The Gharbi and WHO-IWGE classifications are summarized in Table 1.

### 2.6. Clinical Monitoring

Clinical monitoring involved assessing patients who presented with hepatobiliary symptoms such as abdominal pain, a mass in the upper right quadrant, and jaundice using ultrasonography, as is standard practice worldwide, including at our clinic. Indirect hemagglutination (IHA) tests were conducted on all patients, and ultrasound examinations were performed as well. Not all tests yielded positive results. However, all diagnoses were made using ultrasonography. All patients were diagnosed with *E. granulosus*. In patients where simple cysts or hydatid cysts of the liver were identified through ultrasonography, indirect hemagglutination tests, complete blood count parameters, and liver function tests were conducted. For cases where a definitive diagnosis of hydatid cyst could not be made through ultrasonography (i.e., when differentiation from simple cysts or solid lesions was not possible), computed tomography (CT) scans were performed. Following the detection of liver hydatid disease, additional organ screenings were carried out, such as chest radiography (or chest CT if necessary) for pulmonary hydatid disease and renal ultrasonography for renal hydatid disease.

**Figure 1:**
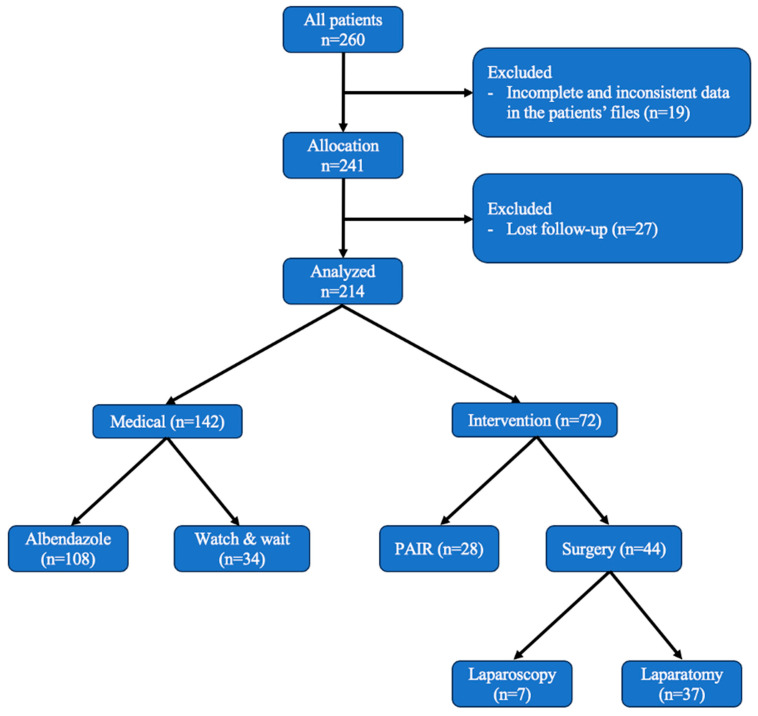
Flow chart of patient selection.

All patients diagnosed with liver hydatid disease were treated according to the WHO IWGE guidelines [16,17,18]. Patients with CE4 and CE5 types were not treated but were monitored through a watch-and-wait approach every 2–4 months. Patients undergoing medical treatment were started on a 6–12-month course of albendazole treatment, following a protocol of 3 weeks on treatment followed by 1 week off. Patients were recalled for clinic visits every 2–4 months, where they underwent a medical history review, physical examination, liver function tests, and ultrasonography. Patients who developed cyst rupture during follow-up were admitted for conservative treatment. Cysts incidentally found during other surgeries were not intervened upon but were instead followed up according to the protocol. Symptomatic patients showing progression despite albendazole treatment were considered for PAIR or surgery as needed [19]. Pre-surgical albendazole treatment was administered for 1–3 weeks to patients planned for PAIR or surgery, which was continued for 1–3 months post-surgery. In cases where there was a high risk of recurrence, this treatment was extended up to 6 months (Figure 1).

### 2.7. Statistical Analysis

Descriptive statistics for all categories, including frequencies and other characteristics of patient data, were conducted. Continuous data were expressed as mean ± standard deviation. The normal distribution of the data was assessed using the Shapiro–Wilk and Kolmogorov–Smirnov tests for continuous variables. Continuous and normally distributed variables were compared using the Student’s *t*-test. Non-parametric tests were selected for data not normally distributed. Categorical variables were compared using the Chi-square test. Analyses were performed using SPSS Statistics for Windows, Version 24.0 (IBM Corp., Armonk, NY, USA). All *p*-values were two-tailed, and values of *p* ≤ 0.05 were considered statistically significant.

## 3. Results

**Figure 2:**
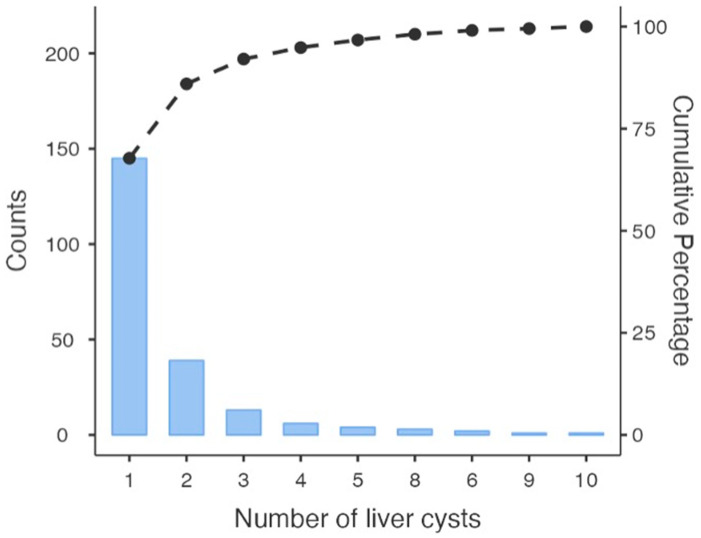
Distribution of liver hydatid cysts (number of observed cysts in liver).

In our study, we included a total of 214 patients, of whom 56% (*n* = 119) were female. The average age of the patients was 11.1 ± 3.8 (range: 1–18) years. We detected a total of 361 hydatid cysts of the liver in our study. Based on the findings, the distribution of cysts was as follows: 46% were classified as CE 1, indicating the initial stage of cyst development. A smaller portion, 11%, fell into the CE 2 stage. CE 3A and CE 3B accounted for 15% and 12% of the cysts, respectively. Furthermore, 11% of the cysts were identified as CE 4, indicative of a more severe and complicated stage. Lastly, a minimal 5% of the cysts were categorized as CE 5 (Table 1). Among the patients, 68% (*n* = 145) had a single cyst, 18% (*n* = 39) had two cysts, 6% (*n* = 13) had three cysts, and 8% (*n* = 17) had four or more liver cysts. A pareto chart graph is given in Figure 2.

In our patient cohort, 86% (*n* = 184) were found to have isolated liver cysts. Additionally, liver and lung involvement was observed in 20 patients, liver and spleen in 5 patients, liver and intraabdominal areas in 2 patients, liver and kidney in 1 patient, liver along with kidney and brain in another patient, and 1 patient had liver and rectus muscle cysts identified (Table 2). Out of 214 patients, 66% were managed non-operatively. Surgery was performed in 34% of cases, with PAIR in 13%. In surgery group, laparoscopy was conducted in 16%, laparotomy in 84%, and one case required conversion to laparotomy (Table 3).

Elevated eosinophils were found in 55% of patients, direct bilirubin in 37%, ALT in 28%, AST in 22%, GGT in 17%, and amylase in 5%. No significant statistical difference was observed between Group 1 and Group 2 in terms of age, gender, and basic laboratory values (*p* > 0.05 for each comparison). When comparing general characteristics of the cysts, such as the lobe where the cyst was located, involvement of multiple organs, number of cysts, the state of cyst rupture, and recurrence, no statistically significant difference was found between the groups (*p* > 0.05 for each comparison). The average cyst size in Group 1 was found to be 60 ± 30.8 mm, while in Group 2, it was 100 ± 30.5 mm, indicating a statistically significant difference in cyst size between the groups (*p* = 0.000). In Group 1, 51% of the cysts were <5 cm, 45% were between 5–10 cm, and 4% were >10 cm. In Group 2, no cysts <5 cm were detected, with 46% of cysts between 5–10 cm and 54% >10 cm (*p* = 0.000). The incidence of solitary organ involvement was found to be 86%, solitary cyst incidence was 68%, cyst rupture incidence was 6%, and the average incidence of recurrence was 2%, with a surgical recurrence incidence of 3%. The average follow-up period was 72 months, during which no mortality was observed (Table 4).

In a patient who underwent PAIR with a drain inserted into the cyst, due to ongoing bile leakage for three months, an Endoscopic Retrograde Cholangiopancreatography (ERCP) with sphincterotomy was performed. Concurrently during the ERCP procedure, a nasobiliary drain was placed. The patient benefited from the nasobiliary drainage, with the bile leakage gradually decreasing and eventually ceasing. Forty-four patients underwent surgery. Of these, 37 had a laparotomy, while 7 had laparoscopic surgery. In one of the laparoscopic cases, the procedure was converted to laparotomy due to perioperative anaphylaxis secondary to the cyst. In total, capitonnage was performed in 68 patients, omentopexy in 4, and cystostomy in 6. Two patients who underwent open surgery experienced bile leakage for about three months postoperatively. One of these patients had previously undergone surgery for gastroschisis and cholecystectomy and required a repeat laparotomy. The other patient underwent ERCP. Consequently, among the treated patients (PAIR + surgery), the incidence of cysto-biliary fistula was 11%, anaphylaxis was 2%, surgical recurrence was 3%, and the incidence of reoperation (Clavien–Dindo ≥ 3) was 6%. In all patients who developed a cysto-biliary fistula and patients who required ERCP, the cysts were located in the right lobe, and the cyst sizes were greater than 10 cm and multicystic (Table 5).

## 4. Discussion

Our study provides of hepatic hydatid cysts in a pediatric population, focusing on the distribution of cyst stages, treatment outcomes, and associated complications. We examined the effectiveness of both surgical and non-surgical treatments, identifying important clinical outcomes such as cysto-biliary fistula formation, recurrence, and post-operative complications.

The liver remains the most commonly affected organ in about 75% of cases, followed by the lungs and spleen. Despite recent advancements in diagnostic methods and medical and interventional treatments, surgery continues to be the cornerstone of management. A range of surgical techniques from aspiration to radical resection has been advocated [2,15,19].

In a study conducted by Öztorun et al., (2021), a total of 205 patients with hydatid cysts were examined, finding that 169 patients (approximately 75%) had liver hydatid cysts, making isolated hepatic hydatidosis the most common presentation [20]. The most frequent concomitant condition was pulmonary hydatid cysts, occurring in 20% of cases. In contrast, a study by Sheves et al., (2023) in Israel identified 70% of liver hydatid cysts as isolated [21], while Mandal et al., (2022) case series in India the same year reported an incidence of 83% for isolated hepatic hydatid cysts [22]. Öztürk et al., (2006) found a slightly lower incidence of 67% [23]. Another study in India by Pradhan et al., (2022) focused on pediatric cases, revealing that 78% of all cases had liver-localized hydatid cysts, with half of the cases presenting the liver as the sole site of involvement [24]. Co-infection of liver and lungs was observed in 21.4% of cases, and a unique case of liver involvement with an additional pelvic hydatid cyst was found in 7.1% of patients. Our study identified 361 hydatid cysts in the liver among 214 patients, with 68% having a single cyst, 18% two cysts, 6% three cysts, and 8% four or more cysts. Isolated liver cysts were present in 86% of patients. Additionally, complex involvements including liver plus lung in 20 patients, liver plus spleen in 5, liver plus intraabdominal (pelvic) in 2, liver plus kidney in 1, liver plus kidney plus brain in 1, and liver plus rectus muscle cyst in another patient were identified. Our study found a higher incidence of isolated hepatic hydatidosis compared to the aforementioned studies.

Tüz et al., (2022) analyzed 57 cases, finding an average cyst size of 66 mm [25]. Sheves et al., (2023), examined 66 cases and reported an average cyst size of 68 mm [21]. Masood et al., (2022), in their study of 60 cases, found the average cyst size to be 88 mm [26]. Öztorun et al., (2021) studied 205 cases and determined an average cyst size of 86 mm [20]. Öztürk et al., (2006)’s study involving 49 cases, identifying an average cyst size of 105 mm [23]. Aygün et al., (2020)’s study of 56 cases reported an average cyst size of 60 mm [27]. Ran et al., (2020) compared patients undergoing radical and conservative surgery across 112 cases, with an average cyst size of 86 mm identified [11]. Our study found that 33.6% of cysts (*n* = 72) were small (<5 cm), 45.3% were medium-sized (5–10 cm), and 21.1% were large (>10 cm), with an average cyst size of 66.97 ± 34.23 mm (median = 60; range: 8–180). The results across various studies show variations in average cyst sizes, which could be attributed to differences in geographic locations, patient populations, and methodologies. Our findings are consistent with the trajectory observed in other studies within the literature.

Tural-Kara et al., (2018) found that 45% of liver hydatid cysts were located in the right lobe, 40% in the left lobe, and 15% in both lobes [28]. Tüz et al., (2022)’s research further revealed that 52% of the cysts were classified as type 1, 14% as type 2, 26% as type 3, and 4% as types 4 and 5, with over 50% of cysts predominantly found in the right lobe and 28% in the left [25]. Mandal et al., (2022) recorded 83% of hydatid cysts in the liver’s right lobe [22]. Additionally, Masood et al., (2022) conducted a randomized controlled trial comparing laparoscopic and open surgical treatments for liver hydatid disease, finding that 67% of cysts were located in the right lobe and 70% were type CE 1 cysts [26]. In our study, we analyzed the classification of cysts at the time of presentation according to the WHO classification. Our results show that 46% of cysts were CE 1, 11% were CE 2, 15% were CE 3A, 12% were CE 3B, 11% were CE 4, and 5% were CE 5. Cysts were found on the right side in 66.8% of patients, on the left side in 21%, and on both sides in 12.1% of cases. The findings of our current study phase align with previous research in the literature, reinforcing the accuracy of the existing knowledge and methods. It should also be noted that the number of patients included in our study is higher compared to those reported in the literature, which enhances the reliability of our study and its potential to make a substantial contribution to the accepted body of knowledge.

Studies in the literature report that eosinophilia occurs in 20% to 34% of hydatid disease cases and may be associated with cyst rupture, particularly in complicated cysts where eosinophilia is a significant laboratory finding [5,12,13,14]. While diagnosis of hydatid disease primarily relies on radiological imaging, epidemiological data, and clinical findings also contribute to the confirmation of the diagnosis. The combination of these methods results in a high detection rate for the disease [12,13]. In a study published in 2022 by Tüz et al. which shared a decade of experience, eosinophilia was found in 39% of patients [25]. In our study, laboratory tests revealed eosinophilia in 55%, elevated direct bilirubin in 37%, ALT in 28%, AST in 22%, GGT in 17%, and amylase in 5% of patients.

The incidence of cyst rupture in hepatic hydatid disease is reported at varying rates in the existing literature, influenced by factors such as the study population, regional factors, cyst size, and the clinical characteristics of the patients. Generally, the incidence of cyst rupture is reported to range between 5% and 40% [2,18,25,29]. Cyst rupture is one of the most significant and dangerous complications of hepatic hydatid cysts, potentially spreading to the peritoneal cavity, biliary system, or adjacent organs. This can lead to severe allergic reactions, sepsis, shock, and even death in patients [18,29,30]. Tüz et al., (2022) reported the incidence of cyst rupture at 18% [25]. In another study, the incidence of hydatid cyst rupture was noted to be 16%, with patients presenting with rupture experiencing prolonged symptoms [31]. Aygün et al., (2020) identified a rupture incidence of 21% in their patients [27]. In our study, cyst rupture occurred in 6% of patients, who were then hospitalized and monitored conservatively.

Adjunctive treatment with albendazole and PAIR has demonstrated fewer complications, higher cure rates, and lower recurrence rates compared to surgery alone, making it a frequently used approach in hepatic hydatid cysts [1,2,32]. In a study, 46 patients were included, with 38% receiving albendazole monotherapy [21]. The same study reported that 98% of patients received albendazole in combination with either PAIR or surgery, and 40% underwent surgery, with a 3% incidence of anaphylaxis [21]. Öztorun et al. reported a surgical incidence of 65% and a PAIR incidence of 35%, with a recurrence rate of 6.5% and a cysto-biliary fistula rate of 7.5% [20]. Öztürk et al. found that 96% of patients underwent surgery, 4% received PAIR, and the recurrence rate was 4% [23]. Karabulut et al. reported that out of 114 patients, 72% underwent surgery and 28% received percutaneous treatment, with 5% experiencing recurrence, 3% cyst infection, and 3% prolonged bile drainage [33]. In another study by Aygün et al., 41 patients were included, with 37% undergoing surgery plus medical treatment and 10% receiving PAIR plus medical treatment [27]. The study reported a 6% incidence of cysto-biliary fistula and a 3% incidence of anaphylaxis. In a study involving only surgical patients, the recurrence rate was 3% and the cysto-biliary fistula rate was 6%. Tüz et al. reported that 78% of patients underwent surgery and 9% received PAIR, with a recurrence rate of 9% [25]. Kaman et al. found that 49% of patients underwent surgery, 23% received PAIR, and the recurrence rate was 8% [31]. Surgical treatment rates in the literature vary between 25–80%, with surgical recurrence rates of 0–10%, cysto-biliary fistula rates of 0–7%, and anaphylaxis rates of 0–7%. The incidence of PAIR appears to vary depending on the availability of interventional radiology in different centers. In our study, 66% (*n* = 142) of patients were observed non-operatively, with 76% receiving albendazole and 24% under observation without treatment (watch-and-wait). Excluding watch-and-wait patients (CE 4 and CE 5), 180 patients required treatment, with 40% undergoing surgery and 60% receiving non-operative treatment. Twenty-eight patients underwent PAIR; one patient who did not benefit from PAIR later underwent open surgery, and another patient received PAIR twice over a year. Forty-four patients underwent surgery (laparoscopy = 7, laparotomy = 37). In our center, the incidence of cysto-biliary fistula was 22%, anaphylaxis 2%, and surgical recurrence 3%. Our findings are consistent with the literature, although our surgical incidence appears lower.

Our study, while comprehensive, has certain limitations. The retrospective design may introduce selection bias and limit the analysis to pre-existing data, making it challenging to establish causality. The reliance on hospital records may also lead to information bias due to potential inaccuracies or omissions in the data. Although we achieved a large sample size, this represents a single-center experience, which may not be generalizable to other regions or populations. Furthermore, the predictive models developed for cyst size and number, while novel, require validation in prospective, multicenter studies to confirm their utility and accuracy. This study’s time frame may also affect the relevance of the findings due to changes in diagnostic and treatment practices over time.

## 5. Conclusions

Our study provides valuable insights into the complications associated with hepatic hydatid cysts in a pediatric population based on a 13-year experience at a high-volume hospital. We identified key clinical outcomes related to both non-surgical treatments (cyst rupture and recurrence) and surgical groups (cysto-biliary fistulas, anaphylaxis, the need for reoperation, rupture, and recurrence). While there were no significant differences in basic clinical characteristics between treatment groups, this study highlighted the greater occurrence of complications in larger and multicystic cases, particularly in those requiring invasive procedures. The recurrence rates were low, and no mortality was observed during the follow-up period.

## Figures and Tables

**Table 1:** Classifications of liver hydatid cysts.

WHO-IWGE	Definition	Gharbi	Stage	*n* (%)
CE1	Unilocular unechoic cystic lesion with double line sign	I	Active	99 (46%)
CE2	Multiseptated, rosette-like, honeycomb cyst	III	Active	23 (11%)
CE3A	Cyst with detached membranes (water-lily-sign)	II	Transitional	32 (15%)
CE3B	Cyst with daughter cysts in solid matrix	III	Transitional	26 (12%)
CE4	Cyst with heterogenous hypoechoic/hyperechoic contents. No daughter cysts	IV	Inactive	24 (11%)
CE5	=CE4 plus calcified wall	V	Inactive	10 (5%)

WHO: World Health Organization, CE: cystic echinococcus.

**Table 2:** Organs with concurrent cyst localization.

Cyst Localization	*n*	%
Isolated Liver	184	86%
Liver + Lung	20	9.3%
Right	14	
Left	4	
Bilateral	2	
Liver + Spleen	5	2.3%
Liver + Intraabdominal	2	0.9%
Liver + Kidney	1	0.5%
Liver + Rectus Muscle	1	0.5%
Liver + Kidney + Brain	1	0.5%
Total	214	100%

**Table 3:** Treatment strategy of the liver hydatid cysts.

	*n*	% *	% ^
Non-operative	142	66%	100%
Medical treatment (Albendazole)	108	50%	76%
Observation without treatment	34	16%	24%
Operative (+Albendazole)	72	34%	100%
PAIR	28	13%	39%
Surgery	44	21%	61%
Laparoscopy	7		16%
Laparotomy	37		84%
Conversion to laparotomy	1		

PAIR: Puncture, Aspiration, Injection, and Re-aspiration; * percentage of intergroups, ^ percentage of intragroups.

**Table 4:** Comparison of the groups.

* (μ ± σ), ^#^ (*n*, %)		Group 1 (*n* = 142)		Group 2 (*n* = 72)		Total (*n* = 214)		*p*
Demographics	Age (year) *	10.8 ± 3.8		12.03 ± 3.53		11.05 ± 3.83		>0.05
	Gender ^#^							>0.05
	Female	80	56%	42	58%	122	56%	
	Male	62	44%	30	42%	92	44%	
Laboratory results	Eosinophil (1 × 10^3^/uL) *	1.27 ± 0.93		1.28 ± 0.89		1.27 ± 0.93		>0.05
	Bilirubin (mg/dL) *	0.35 ± 0.21		0.37 ± 0.21		0.35 ± 0.21		>0.05
	Amylase (U/L) *	72.22 ± 29.65		76.35 ± 33.34		73.24 ± 31.13		>0.05
	ALT (U/L) *	34.47 ± 29.81		40.31 ± 32.65		36.65 ± 31.57		>0.05
	AST (U/L) *	44.86 ± 36.33		45.12 ± 37.98		44.97 ± 37.12		>0.05
	GGT (U/L) *	56.88 ± 32.37		58.36 ± 33.03		57.13 ± 32.94		>0.05
Feature of the cysts	Localization ^#^							>0.05
	Right lobe	95	67%	48	66%	143	67%	
	Left lobe	30	21%	15	21%	45	21%	
	Bilateral	17	12%	9	13%	26	12%	
	Organ Involvement ^#^							>0.05
	Isolated in liver	119	84%	65	90%	184	86%	
	Multiple organs	23	16%	7	10%	30	14%	
	Number of Cysts ^#^							>0.05
	Single	95	67%	50	69%	145	68%	
	Multiple	47	33%	22	31%	69	32%	
	Cyst Diameter ^#^							0.000
	<5 cm	72	51%	0	0%	72	34%	
	5–10 cm	64	45%	33	46%	97	45%	
	>10 cm	6	4%	39	54%	45	21%	
	Cyst diameter (mm) *	60.1 ± 30.8		100 ± 30.5		66.9 ± 34.2		0.000
	Cyst Rupture ^#^							>0.05
	No	135	95%	66	92%	201	94%	
	Yes	7	5%	6	8%	13	6%	
	Recurrence ^#^							>0.05
	No	140	99%	70	97%	210	98%	
	Yes	2	1%	2	3%	4	2%	

* μ ± σ: mean ± standart deviation, ^#^ (*n*, %), ALT: alanine aminotransferase; AST: aspartate aminotransferase; GGT: gamma glutamyl transferase.

**Table 5:** Analysis of the complications of the cysts which underwent surgery or PAIR.

Complications	*n*	%
Anaphylaxis	1	2%
Cysto-biliary fistula	8	11%
Need for ERCP	2	3%
Nasobiliary catheter insertion	1	2%
Clavien–Dindo classification (≥3)	4	6%

ERCP: Endoscopic Retrograde Cholangiopancreatography.

## Data Availability

The datasets generated during and/or analyzed during the current study are available from the corresponding author on reasonable request.
